# The Role of SIRT3 in the Brain Under Physiological and Pathological Conditions

**DOI:** 10.3389/fncel.2018.00196

**Published:** 2018-07-25

**Authors:** Elena Sidorova-Darmos, Rosa Sommer, James H. Eubanks

**Affiliations:** ^1^Division of Genetics and Development, Krembil Research Institute, University Health Network, Toronto, ON, Canada; ^2^Department of Physiology, University of Toronto, Toronto, ON, Canada; ^3^Institute of Medical Sciences, University of Toronto, Toronto, ON, Canada; ^4^Department of Pharmacology and Toxicology, University of Toronto, Toronto, ON, Canada; ^5^Department of Surgery (Neurosurgery), University of Toronto, Toronto, ON, Canada

**Keywords:** SIRT3, mitochondria, oxidative stress, neurodegenerative disease, pharmacology

## Abstract

Sirtuin enzymes are a family of highly seven conserved protein deacetylases, namely SIRT1 through SIRT7, whose enzymatic activities require the cofactor nicotinamide adenine dinucleotide (NAD^+^). Sirtuins reside in different compartments within cells, and their activities have been shown to regulate a number of cellular pathways involved in but not limited to stress management, apoptosis and inflammatory responses. Given the importance of mitochondrial functional state in neurodegenerative conditions, the mitochondrial SIRT3 sirtuin, which is the primary deacetylase within mitochondria, has garnered considerable recent attention. It is now clear that SIRT3 plays a major role in regulating a host of mitochondrial molecular cascades that can contribute to both normal and pathophysiological processes. However, most of the currently available knowledge on SIRT3 stems from studies in non-neuronal cells, and the consequences of the interactions between SIRT3 and its targets in the CNS are only beginning to be elucidated. In this review, we will summarize current advances relating to SIRT3, and explore how its known functions could influence brain physiology.

## Introduction

It has been over three decades since the silent information regulator 2 (*Sir2*) gene was initially identified in yeast (Shore et al., 1984; Ivy et al., 1986; Rine and Herskowitz, 1987; Tanner et al., 2000) and its product shown to regulate DNA recombination/repair, gene silencing and chromosomal stability (Brachmann et al., 1995; Kaeberlein et al., 1999). Interest in SIR2 increased substantially when longevity-enhancements were associated with its over-expression in *Saccharomyces cerevisiae* (Kaeberlein et al., 1999; Kim et al., 1999; Wood et al., 2004), *C. elegans* (Tissenbaum and Guarente, 2001) and *Drosophila melanogaster* (Wood et al., 2004), and the identification of seven SIR2 homologs (SIRT1–7) in mammalian systems (Brachmann et al., 1995; Frye, 2000; Finkel et al., 2009) only added further interest to the family. Unlike the restricted nuclear localization of the ancestral SIR2 protein in yeast, however, specific mammalian sirtuins localize within different sub-cellular compartments. Like the ancestral Sir2, sirtuins SIRT1, SIRT6 and SIRT7 reside predominantly within the nucleus (Michishita et al., 2005). SIRT2 is largely cytosolic (Perrod et al., 2001; North et al., 2003), while sirtuins SIRT3, SIRT4 and SIRT5 are primarily found within mitochondria (Onyango et al., 2002; Ahuja et al., 2007; Nakamura et al., 2008). Defining the role played by each individual sirtuin within these specific regions remains a major topic of investigation. Structurally, while each member of the sirtuin family possesses a conserved catalytic core domain and a nicotinamide adenine dinucleotide (NAD^+^) binding motif, each member displays distinct N- and C-terminal regions that contribute to their localization and functional specificities (Landry et al., 2000; Min et al., 2001; Marmorstein, 2004). While other enzymatic activities have been reported for some members of the family (see Table 1, Du et al., 2011; Peng et al., 2011; Jiang et al., 2013; Bao et al., 2014; Mathias et al., 2014; Tan et al., 2014), NAD^+^-dependent protein deacetylase and/or as a mono-[ADP-ribosyl] transferase activity appears to be the primary enzymatic function for the different sirtuins (Frye, 1999; Schwer et al., 2002; North et al., 2003; Liszt et al., 2005; Michishita et al., 2005; Mostoslavsky et al., 2006; Ahuja et al., 2007; Schlicker et al., 2008; Vakhrusheva et al., 2008; see Table 1). Due to their dependence on intracellular NAD^+^ levels, the family of sirtuins has been hypothesized to act as key rheostats that collectively monitor and maintain the homeostatic balance of the intracellular environment (Kupis et al., 2016).

**Table 1:** Mechanisms of action for sirtuin family members.

Sirtuin	Catalytic Activities
SIRT1	Deacetylase (Michishita et al., 2005),
	Decrotonylase (Bao et al., 2014)
SIRT2	Deacetylase (Perrod et al., 2001; North et al., 2003),
	Decrotonylase (Bao et al., 2014)
SIRT3	Deacetylase (Schwer et al., 2002),
	Decrotonylase (Bao et al., 2014)
SIRT4	ADP-ribosyltransferase (Ahuja et al., 2007),
	Lipoamidase (Mathias et al., 2014)
SIRT5	Deacetylase (Schlicker et al., 2008)
	Demalonylase (Du et al., 2011; Peng et al., 2011),
	Desuccinylase (Du et al., 2011; Peng et al., 2011)
	Glutarylate (Tan et al., 2014)
SIRT6	ADP-ribosyltransferase (Liszt et al., 2005);
	Deacetylase (Michishita et al., 2008),
	Acylate (Jiang et al., 2013)
SIRT7	Deacetylase (Vakhrusheva et al., 2006, 2008)

Perturbations in mitochondrial homeostasis are hypothesized to play pathogenic roles in a number of neurological diseases and conditions (Martin, 2012; Morris and Berk, 2015). Normal mitochondrial homeostasis is achieved by maintaining the proper complement and levels of mitochondrial proteins and substrates, and by governing the functional activity of these proteins by post-translational modification (Stram and Payne, 2016). One of the most common protein post-translational modifications in the mitochondria proteome is lysine acetylation, with over 65% of mitochondrial proteins being subject to acetyl-lysine regulation (Hebert et al., 2013). The SIRT3 sirtuin plays a key role in this regulation, as altered mitochondrial proteome hyperacetylation has been clearly observed in tissues from SIRT3 knockout (KO) mice, with little to no changes in mitochondrial protein acetylation observed in the same tissues from SIRT4 or SIRT5-KO mice (Lombard et al., 2007). As post-translational protein acetylation is a modification that can dramatically influence the enzymatic activity of target proteins, these data support the hypothesis that SIRT3 serves as a key rheostat that maintains cellular metabolic homeostasis by altering the acetylation state of the mitochondrial proteome in response to changes in cellular demands (Hirschey et al., 2010; Tao et al., 2010; Cheng et al., 2016). As the brain has a particularly high aerobic metabolic rate and consumes proportionally large amounts of ATP (Falkowska et al., 2015), it is understandable why there is an increasing interest to better understand the regulation of SIRT3 expression and functional activity in nervous system tissues.

## SIRT3 Expression in the Brain

Expression analysis has revealed SIRT3 to be preferentially expressed in tissues with high oxidative capacity such as brain, cardiac, hepatic, brown adipose tissue and skeletal muscle, with lower levels of expression found in tissues with lower-metabolic demand such as white adipose tissue, lungs, spleen, thymus, pancreas and small intestine (Shi et al., 2005; Lombard et al., 2007; Palacios et al., 2009; Sidorova-Darmos et al., 2014; Braidy et al., 2015). At the mRNA level, *Sirt3* is the third highest expressed of all the sirtuins in the adult rat brain (behind *Sirt2* and *Sirt5*; Sidorova-Darmos et al., 2014). High expression of SIRT3 in the CNS is not surprising, given its role in regulating mitochondrial function, metabolism and in regulating oxidative defense systems. At the protein level, SIRT3 is expressed at similar levels in the adult rat cortex, hippocampus, striatum, spinal cord, and brain stem, but is expressed at lower levels in the cerebellum (Sidorova-Darmos et al., 2014). The lower SIRT3 protein levels in cerebellum may relate to the lower number of mitochondria present in cerebellum relative to other brain regions (Frahm et al., 2005; McInerny et al., 2009; Fuke et al., 2011). SIRT3 expression within the brain is not restricted to neurons, as clear SIRT3 expression has been demonstrated in astrocytes and microglia (Kim et al., 2011; Weir et al., 2012; Dai et al., 2014; Sidorova-Darmos et al., 2014; Cheng et al., 2016). However, the relative prevalence of SIRT3 is higher in cortical neurons than in astrocytes at both the mRNA and protein levels (Sidorova-Darmos et al., 2014).

The expression profile of SIRT3 in the brain is also developmentally regulated. At the mRNA level *Sirt3* expression is fairly consistent from embryonic day 18 until 24 months of age in the rat cortex, cerebellum and hippocampus (Sidorova-Darmos et al., 2014). A different expression pattern is seen for SIRT3 protein during development, however, as in the rat cortex, hippocampus and cerebellum, SIRT3 protein levels increase significantly between post-natal day 7 and post-natal day 21 (Sidorova-Darmos et al., 2014). Because mitochondria play a central role in energy supply and are major regulators of neural development (Xavier et al., 2016), the observed elevation in SIRT3 levels correlates with times of increased energy demand in the central nervous system due to cellular/synaptic growth and maturation. Studies examining SIRT3 expression levels in aged brains have yielded somewhat conflicting results, however. A decrease in SIRT3 protein levels was reported in the 18 month-old rat auditory cortex relative to 4 month-old rats (Zeng et al., 2014), and in 24 month-old rat hippocampus and frontal lobe relative to the same regions in three month-old rats (Braidy et al., 2015). However, Sidorova-Darmos et al. (2014) reported SIRT3 levels remain relatively consistent between postnatal day 21 and 24 months in the rat hippocampus and cortex, and that its expression levels increase modestly in the cerebellum between these times. The reason for this seeming discrepancy is not clear, and could potentially relate to strain and/or housing condition differences. Collectively though, these studies do show that SIRT3 is expressed in different cell lineages of the brain from early development and maintained until elderly stages, and therefore allow one to explore how drugs selectively targeting SIRT3 could influence brain function throughout life.

## SIRT3 Protein Is Expressed as a Precursor and Trafficked to Mitochondria

Assessments of endogenous SIRT3 localization have largely demonstrated a mitochondrial residence for SIRT3 in cells from different species (Schwer et al., 2002; Lombard et al., 2007; Scher et al., 2007). SIRT3 is expressed initially as a precursor protein of approximately 45 kilodaltons that contains a mitochondrial localization sequence (Onyango et al., 2002). Upon entry into the mitochondrial matrix, this pro-form of SIRT3 is processed into a shorter form by mitochondrial matrix processing peptidase (MMP; Onyango et al., 2002; Schwer et al., 2002). Recently, it was reported that this MMP-generated product is further cleaved by mitochondrial intermediate peptidase (MIPEP) to generate the mature enzymatically active 28 kilodalton form of SIRT3 present in the mitochondrial matrix (Kobayashi et al., 2017). However, while ubiquitously agreed that SIRT3 does reside within mitochondria, there remains debate regarding whether or not a population of some form of SIRT3 may also reside in nucleus or cytoplasm (Scher et al., 2007; Nakamura et al., 2008; Sundaresan et al., 2008). Indeed, nuclear localization of the endogenous SIRT3 precursor has been reported in U2OS and HeLa cells by immunocytochemical analysis (Scher et al., 2007; Iwahara et al., 2012) and both nuclear localization and nuclear deacetylase activity has been reported in HeLa cells and rat cardiomyocytes ectopically expressing a full-length human *SIRT3* cDNA (Scher et al., 2007; Sundaresan et al., 2008). However, Cooper and Spelbrink (2008) failed to detect any nuclear SIRT3 using stringent subcellular fractionation techniques in HeLa, U2OS or HEK-293 cells that ectopically expressed a full-length human *SIRT3* cDNA. Onyango et al. (2002) reported no detectable nuclear signal in COS7 cells ectopically expressing a SIRT3-GFP reporter system and a lack of endogenous SIRT3 was observed in nuclear extracts isolated from rat skeletal muscle (Gurd et al., 2012). Thus, while it is clear that the processed form of catalytically active SIRT3 resides within the mitochondria where it targets a number of mitochondrial proteins (reviewed in Bell and Guarente, 2011; Hirschey et al., 2011; Bause and Haigis, 2013; Ansari et al., 2017), it remains controversial whether additional subcellular expression patterns and non-mitochondrial protein targets exist for SIRT3 under normal conditions.

## SIRT3 Function in Mitochondria

SIRT3 is the primary sirtuin deacetylase in mitochondria. SIRT3-KO mice display globally elevated mitochondrial acetylation levels in liver, while SIRT4 or SIRT5 KO mice show no changes in mitochondrial proteome acetylation (Lombard et al., 2007). Moreover, a hyperacetylation of mitochondrial proteins was observed in myocardial tissue following infarction, and this change coincided with a downregulation of SIRT3 expression with both SIRT4 and SIRT5 expression levels being unaffected (Parodi-Rullan et al., 2012). It is important to note, however, that the role of SIRT4 and SIRT5 in mitochondria can provide benefit independent from SIRT3. For example, over-expression of SIRT4 markedly protected H9c2 cardiomyoblast cells from apoptosis following hypoxia (Liu et al., 2013), and SIRT5 over-expression in SH-EP neuroblastoma cells decreased apoptosis and oxidative stress induced by staurosporine and hydrogen peroxide, respectively (Liang et al., 2017). While a substantial number of mitochondrial SIRT3 substrates have been identified (see below), only a handful of SIRT4 and SIRT5 targets have thus far been uncovered (reviewed in Parihar et al., 2015). This is an area of growing interest, as cooperation between the three mitochondrial sirtuins may be required to maintain proper homeostatic balance within the mitochondria—both under normal and pathogenic circumstances.

While there have been fewer studies of SIRT3 activity in neural cells or tissues, studies employing non-neuronal systems have uncovered a number of mitochondrial proteins targeted by SIRT3 that are also expressed in brain, and therefore it seems likely they will be targeted similarly by SIRT3 in nervous system settings. These include members of pathways involved in anti-oxidative defense systems (Jacobs et al., 2008; Tao et al., 2010; Bell and Guarente, 2011; Chen et al., 2011; Ren et al., 2016), metabolism (Hallows et al., 2006; Ahn et al., 2008; Schlicker et al., 2008; Salvatori et al., 2017), and mitochondrial biogenesis (Shi et al., 2005; Kong et al., 2010; Yang et al., 2010; Giralt et al., 2011; Dai et al., 2014; Zhao et al., 2016).

**Figure 1:**
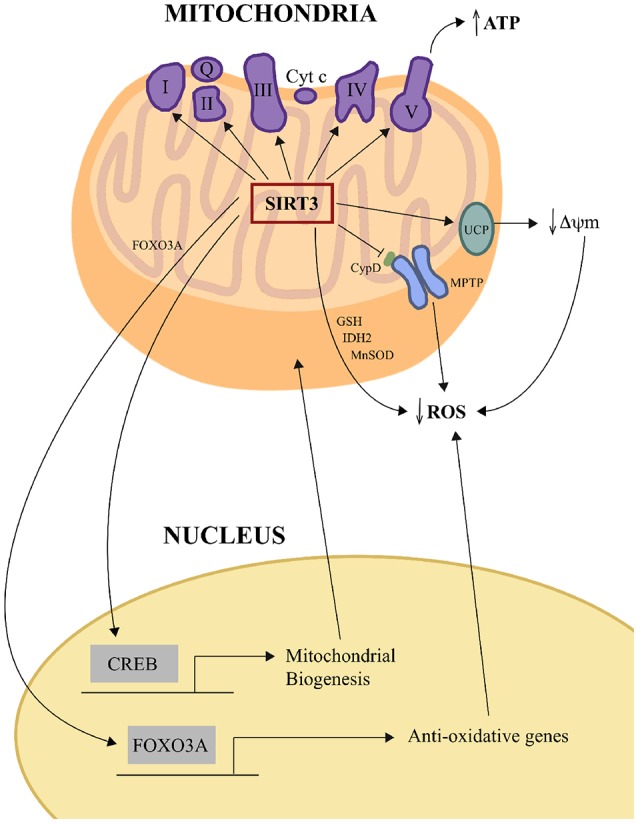
SIRT3 influences oxidative phosphorylation and oxidative stress management. SIRT3 increases ETC efficiency by deacetylating and regulating the activities of mitochondrial complexes I, II, III, IV and V to increase ATP production. SIRT3 reduces mitochondrial oxidative stress directly by deacetylating IDH2 and MnSOD and also increasing GSH levels, and thereby enhancing ROS detoxification. SIRT3 can also negatively regulate ROS levels in some tissues indirectly by upregulating UCP expression, which lessens the driving force for mitochondrial ROS production by decreasing ΔΨm. SIRT3 can also negatively regulate the opening of the mPTP by deacetylating CypD. SIRT3 also orchestrates mitochondria-nuclear cross-talk by deacetylating and activating mitochondrial FOXO3A, which then travels to the nucleus to activate the transcription of anti-oxidative genes, and by indirectly activating the transcription factor CREB, which activates genes whose products play roles in mitochondrial biogenesis. CREB, cAMP response element-binding protein; CypD, cyclophilin D; ETC, electric transport chain; FOXO3a, forkhead box O3; GSH, glutathione; IDH2, isocitrate dehydrogenase; MnSOD, manganese superoxide dismutase; mPTP, mitochondrial permeability transition pore; ROS, reactive oxygen species; UCP, uncoupling protein; ΔΨm, change in mitochondrial membrane potential.

Visual overview of some of these pathways is outlined in Figure 1.

Each of these pathways has direct relevance to brain physiology, and alterations in the function of each have been implicated as contributing factors in different neurodegenerative diseases or neurological disorders (Federico et al., 2012; Guo et al., 2013; Kim et al., 2015; Li et al., 2017).

### SIRT3 Regulation of Anti-Oxidative Defense Systems

Of particular interest to neural cells, SIRT3 deacetylates and increases the catalytic activity of manganese superoxide dismutase (MnSOD; Qiu et al., 2010; Tao et al., 2010; Chen et al., 2011; Shi et al., 2017), which is the primary mitochondrial enzyme that converts O_2_^−^ to H_2_O_2_, and the TCA cycle enzyme isocitrate dehydrogenase (IDH2; Schlicker et al., 2008; Someya et al., 2010; Yu et al., 2012), which converts NADP^+^ to NADPH in mitochondria and helps maintain the anti-oxidant glutathione (GSH) in a reduced and active state. SIRT3 also physically interacts with thioredoxin-2 (Palacios et al., 2009), which is a member of the thioredoxin system and also involved in H_2_O_2_ scavenging. Indeed, converging studies show that cells and tissues deficient in SIRT3 show high indices of oxidative stress (reviewed in Bell and Guarente, 2011; Bause and Haigis, 2013; Ansari et al., 2017). Interestingly Parodi-Rullam et al. (2017) observed no difference in oxidative stress between WT and SIRT3-KO mouse hearts under basal conditions. Because there was an increase in SIRT4 expression in SIRT3-KO hearts, these authors suggested that SIRT4 may serve as an independent anti-oxidative compensatory mechanism in these mice under normal conditions. However, following ischemia-reperfusion injury, SIRT3-deficient heart mitochondria showed elevations of reactive oxygen species (ROS) and oxidative damage relative to WT mitochondria, illustrating any potential compensatory SIRT4 activity has a ceiling relative to that of SIRT3. Future studies will be required to define the interplay between mitochondrial sirtuins and regulation of anti-oxidative systems in mitochondria under basal and various pathological conditions. Nevertheless, these results strongly suggest SIRT3 likely plays a key role in regulating ROS levels in neurons, where aerobic respiration is the primary mechanism for energy production.

### SIRT3 Influence on Mitochondrial Membrane Potential and ROS Genesis

SIRT3 over-expression has been shown to lower mitochondrial membrane potential (ΔΨm) and ROS levels in different non-neuronal cell types (Shi et al., 2005; Bell and Guarente, 2011; Chen et al., 2011; Shulyakova et al., 2014; Liu et al., 2015; Pillai et al., 2016; Ren et al., 2016). While the exact mechanism of how SIRT3 decreases ΔΨm and ROS levels remain to be fully elucidated, a potential mechanism has been suggested for brown adipose tissue (Shi et al., 2005). In these cells, there is a SIRT3-dependent up-regulation of the transcriptional regulator peroxisome proliferator-activated receptor gamma coactivator 1-alpha (PGC1-α) expression, a transcriptional co-activator that regulates the expression of numerous genes involved in mitochondrial function and detoxification pathways (Onyango et al., 2010). PGC1-α facilitates an increase in the expression levels of mitochondrial uncoupling protein 1 (UCP1), which in turn decreases ΔΨm by enhancing proton flux back across the inner mitochondrial membrane into the matrix (Klingenspor, 2003). The lower ΔΨm decreases the driving force for electron leak during oxidative phosphorylation, and thereby contributes to the decrease in ROS genesis (Sullivan et al., 2004). It is unlikely that this direct mechanism would apply to the brain, however, as UCP1 expression is low or undetectable in the CNS under normal conditions (Andrews et al., 2005) and therefore the function of SIRT3 in brain does not likely involve UCP1. It is worth noting though that other relatives of UCP1 are expressed in the brain (UCP2′, -4 and -5)′, and their elevated expression and activity have also been correlated with neuroprotection (Andrews et al., 2005). It is therefore possible that these UCP family members could potentially substitute for UCP1 in neurons as targets for SIRT3. To date, though, this remains speculative, as direct SIRT3 regulation of any UCP family member in neurons remains undetermined.

### SIRT3 Influences the Expression of Nuclear Genes Encoding Anti-Oxidative and Mitochondrial Factors

In addition to its direct activation of antioxidant target proteins by deacetylation, SIRT3 has also been shown to indirectly regulate the expression of specific antioxidant enzymes. Jacobs et al. (2008) showed that the transcription factor forkhead box O3 (FOXO3a) is a substrate of SIRT3 in mitochondria, which upon deacetylation translocates into the nucleus to stimulate the transcription of specific anti-oxidative genes. As stated above, SIRT3 directly or indirectly also influences the expression of PGC-1α. Although the mechanism of SIRT3 regulation of PCG-1α is not fully understood, it has been speculated that SIRT3 indirectly increases the phosphorylation of the transcription factor, cAMP response element-binding protein (CREB; Shi et al., 2005; Kong et al., 2010), which then facilitates PGC-1α gene expression. Thus, SIRT3 regulates mitochondrial proteins at both their expression and functional levels by indirectly regulating their gene-expression in the nucleus, and their catalytic/functional state directly in mitochondria through post-translational deacetylation. These results further illustrate how SIRT3 could be viewed as a key rheostat for maintaining mitochondrial homeostasis.

### SIRT3 Influence on Mitochondrial Permeability Transition Pore Activation

In pathological conditions and following different neural injuries such as stroke or trauma, the formation and opening of the mitochondrial permeability transition pore (mPTP) is a hallmark of end-stage mitochondrial dysfunction that allows for the release of pro-apoptotic factors from mitochondria that activate cell death pathways (Halestrap and Pasdois, 2009; Sims and Muyderman, 2010). Inhibiting mPTP opening has been proposed as a neuroprotective strategy for a number of neurodegenerative diseases (Rao et al., 2014), and one proposed mechanism for SIRT3-mediated cytoprotection involves it inhibiting the opening of the mPTP (Hafner et al., 2010). In cardiac myocytes, Hafner et al. (2010) showed that SIRT3 deacetylase activity inhibited the formation of the mPTP by decreasing the binding of cyclophilin D (CypD) to adenine nucleotide translocase (ANT). The SIRT3-mediated regulation of CypD is evident in brain, as CypD hyperacetylation is observed in hippocampal tissues and cortical neurons isolated from SIRT3-KO mice (Cheng et al., 2016). These results suggest that SIRT3 activity in brain negatively influences mPTP opening by maintaining CypD in a deacetylated state. However, there may be context-specificity or limits to this action of SIRT3, as Novgorodov et al. (2016) found no difference in Ca^2+^-induced mPTP opening in mitochondria isolated from cerebral tissues that were harvested from ischemia-challenged SIRT3-KO mice and wild-type mice. However, the requisite role of acetyl-CypD on mPTP opening may also be context-dependent, as mPTP opening occurred without any CypD acetylation state changes in peroxide-treated H9c2 rat embryonic cardioblasts (Barreto-Torres et al., 2015), and Parodi-Rullam et al. (2017) found no difference in mPTP opening and CypD acetylation in hearts between WT and SIRT3-KO mice following ischemia-reperfusion injury. Further, CypD itself may influence mitochondrial acetylation levels, as mice lacking CypD show hyperacetylation of mitochondrial proteins without any change in SIRT3 expression or activity levels (Nguyen T. T. M. et al., 2013). These studies suggest that the cause-effect between CypD acetylation by SIRT3 and the opening of mPTP is complicated and may be context-dependent. Therefore, future studies are necessary to elucidate the role of SIRT3 on the opening of mPTP under normal physiological and different pathophysiological conditions.

### SIRT3 Influence on Mitochondrial Biogenesis and Mitochondrial Dynamics

SIRT3 has also been implicated in mitochondrial biogenesis regulation (Shi et al., 2005; Kong et al., 2010; Giralt et al., 2011; Dai et al., 2014; Zhao et al., 2016), mitochondrial fusion/fission dynamics (Song et al., 2013; Samant et al., 2014; Morigi et al., 2015), and related aspects of maintaining mitochondrial integrity. Mitochondrial biogenesis is a process through which new mitochondria are generated. This phenomenon is especially important for neural development and may also serve as a protective mechanism during neurodegenerative disease (Onyango et al., 2010). Indeed, studies show that SIRT3 expression is required for proper mitochondrial biogenesis in both mouse C_2_C_12_ myoblasta (Kong et al., 2010) and human oocytes (Zhao et al., 2016). Mitochondrial dynamics also play an important role in the brain, as mitochondria continuously undergo dynamic changes in their size, shape, number, and distribution to appropriately respond to fluctuating metabolic demands (Milone and Benarroch, 2012). Fission results in mitochondrial division, while fusion allows distinct mitochondria to exchange intra-mitochondrial metabolic products and mtDNA. This normal fission/fusion balance can be altered by various external factors, such as ROS elevation (Wu et al., 2011), reduced ATP levels (Mishra and Chan, 2016) and mitochondrial Ca^2+^ influx (Chen and Chan, 2009; Kaddour-Djebbar et al., 2010). Samant et al. (2014) reported that SIRT3 preserves mitochondrial networking in cardiomyocytes by deacetylating and activating the GTPase activity of optic atrophy 1 (OPA1), which is an integral protein involved in promoting mitochondrial fusion. In addition, Morigi et al. (2015) reported that SIRT3 over-expression prevented the mitochondrial recruitment of the fission-promoting protein dynamin-related protein-dependent (DRP1) and also increased OPA1 expression in cultured human renal proximal tubular epithelial cells in response to fission-inducing cisplatin challenge. As mitochondrial fragmentation is evident in many neurodegenerative conditions such as Alzheimer’s disease (AD; Wang et al., 2009), Parkinson’s disease (Van Laar and Berman, 2009), amyotrophic lateral sclerosis (ALS; Jiang et al., 2015), and at early stages following reversible ischemic stroke (Barsoum et al., 2006), it will be of considerable interest to determine whether SIRT3 can be harnessed to preserve mitochondrial integrity in these conditions and circumstances.

### SIRT3 Regulates Energy Source Utilization by Mitochondria

SIRT3-mediated deacetylation and activation of several factors involved in mitochondrial energy production have been demonstrated. These include acetyl coenzyme A synthetase type 2 (AceCS2), glutamate dehydrogenase (GDH), IDH2 type 2, and specific components of the electron transport chain (ETC; Hallows et al., 2006; Schlicker et al., 2008; Someya et al., 2010; Yu et al., 2012). AceCS2 catalyzes formation of acetyl-CoA from free acetate and coenzyme A (CoA), which can then be used via the TCA cycle for ATP synthesis (Fujino et al., 2001). The function of AceCS2 is especially important during periods of diminished glucose availability, as in such times of need the liver can release significant amounts of stored acetate for use by extra-hepatic tissues that include the brain (Yamashita et al., 2001). SIRT3 activation of GDH enhances the production of TCA cycle intermediates α-ketoglutarate and NADH, which enhances the pool of electron donating substrates for oxidative phosphorylation (Schlicker et al., 2008). In this regard, SIRT3 has also been shown to deacetylate and regulate the activity of complex I in general (Kim et al., 2010), and specific mitochondrial respiratory chain complex components such as NDUFA9 of Complex I (Ahn et al., 2008); Succinate Dehydrogenase of complex II (Finley et al., 2011) and ATP synthase subunits α and β (Law et al., 2009; Wu et al., 2013; Rahman et al., 2014). In the absence of SIRT3, the activities complex I, complex II, and complex V were each shown to be decreased relative to control (Ahn et al., 2008; Law et al., 2009; Finley et al., 2011; Rahman et al., 2014; Novgorodov et al., 2016), and the activity of complexes III and IV were also found to be decreased in liver extracts from SIRT3-KO mice maintained on a high fat diet (Kendrick et al., 2011). Consistently, decreased ATP levels were reported in several different organs of SIRT3-KO mice (Ahn et al., 2008). It should be noted, however, that these observations are not absolute, as Fernandez-Marcos et al. (2012) reported no changes in ATP levels in SIRT3-KO mouse muscle or liver, and Novgorodov et al. (2016) reported that complex I activity but not complex II or complex IV activity was reduced under basal conditions in SIRT3-KO mouse cerebral cortex. Interestingly, a decrease in basal mitochondrial oxygen consumption rate has been observed in primary cultured mouse dopaminergic neurons overexpressing SIRT3 (Gleave et al., 2017), suggesting that SIRT3 over-expression increases mitochondrial efficiency by stabilizing respiration at the electron transport level. While these results support SIRT3 as a factor regulating overall cellular energy production at different levels, additional studies are needed to better define how changes in metabolic state affect the activity of SIRT3, and how SIRT3 influences mitochondrial respiratory activity in highly erobic tissues such as brain.

### SIRT3 Influences the Production of Energy Substrates

Brain metabolic demand is regulated by a complex interplay of behavioral state, energy substrate supply and availability, and hormonal signaling between periphery and tissues within the central nervous system (Lenard and Berthoud, 2008). The energy source primarily used by the brain is glucose, and tight regulation of its metabolism is critical for normal brain physiology, structure integrity, and neuronal viability (Mergenthaler et al., 2013; Bauernfeind et al., 2014). However, when glucose availability becomes limited, the brain is able to utilize alternative sources from other sources in the body for energy production (White and Venkatesh, 2011; Prins, 2012; Glenn et al., 2015; Proia et al., 2016). Glycogen reserves can be tapped to maintain blood-glucose levels acutely, and meet the high-metabolic demands of the brain for a short period of time (Waitt et al., 2017). Once these glycogen reserves have been depleted, though, another alternative energy source must serve as substrates. During such conditions, fatty acid reserves from adipose tissues can be catabolized, and used for fatty acid β-oxidation in the liver to generate acetyl-CoA, which is then converted by ketogenesis into ketone bodies (White and Venkatesh, 2011). These ketones are then carried to metabolically active tissues, such as the brain, where they are converted back to acetyl-CoA to serve as an energy source. Studies have suggested SIRT3 plays a vital role in all the major steps of this hepatic “glucose-sparing energy pathway”, as SIRT3 deacetylates and activates two key enzymes involved in fatty acid β-oxidation pathway initiation: long-chain acyl CoA dehydrogenase (LCAD; Hirschey et al., 2010) and very long-chain acyl-CoA dehydrogenase (VLCAD Hallows et al., 2011; Zhang et al., 2015). These interactions have been demonstrated in liver, but it is worth noting that both LCAD and VLCAD are also expressed in brain, and could therefore also be subject to SIRT3 regulation in the CNS. SIRT3 has also been implicated in activating L-3-hydroxyacyl-CoA dehydrogenase, short/branched-chain acyl-CoA dehydrogenase and 3-ketoacyl-CoA thiolase (Hallows et al., 2011), which are each also involved in β-oxidation processes. Moreover, SIRT3 has also been shown to regulate the production of ketone bodies by targeting 3-hydroxy-3-methylglutaryl-CoA synthase (HMGCS2), which is the limiting enzyme in the production of ketone bodies from acetyl-CoA and acetoacetyl-CoA (Shimazu et al., 2010). Intriguingly, SIRT3 not only promotes the synthesis of ketones, it also shifts the hepatic mitochondrial metabolism to meet the higher energy demand that is necessary for ketogenesis. This is accomplished by deacetylating and activating the mitochondrial transcription factor leucine-rich protein 130 (LRP130), who in turn stimulates the transcription of genes in the mitochondrial genome encoding factors required for oxidative phosphorylation cascades (Liu et al., 2011). Whether this occurs similarly in brain as demonstrated in liver remains to be determined.

**Figure 2:**
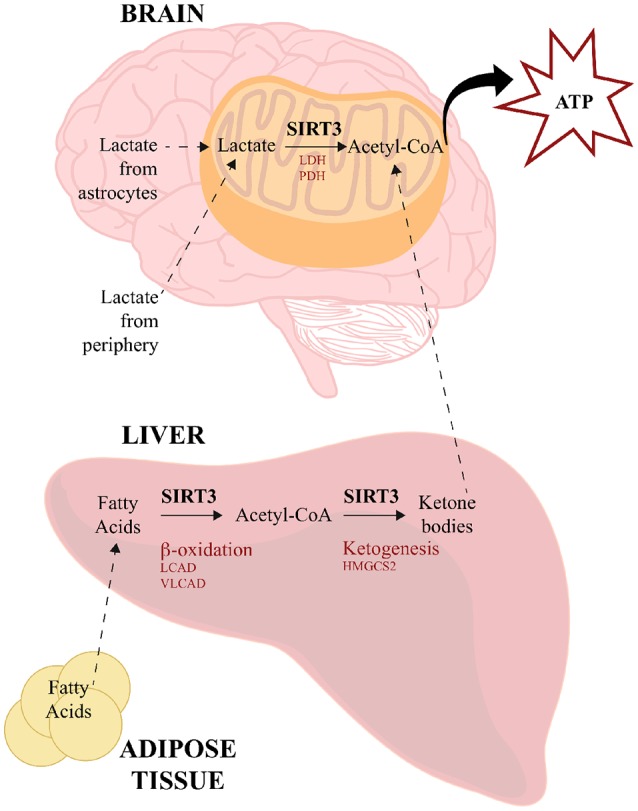
SIRT3 regulates energy source production and utilization. SIRT3 influences the use of alternative sources for energy production to meet high-metabolic demands of the brain. During low glucose availability, fatty acids from adipose tissue are catabolized in the liver via β-oxidation to generate acetyl-CoA, SIRT3 deacetylates and activates LCAD and VLCAD, which are two key enzymes involved in fatty acid β-oxidation. SIRT3 also deacetylates and activates HMGCS2, which is the limiting enzyme in the production of ketone bodies from acetyl-CoA. Ketone bodies travel to the brain, where they are converted back to acetyl-CoA for energy utilization. SIRT3 also deacetylates acetyl-CoA-synthetase 2, which can generate acetyl-CoA from free acetate. Lactate is another energy source derived from peripheral tissues and astrocytes. SIRT3 deacetylates and activates both LDH and PDH, which convert lactate to pyruvate and acetyl-CoA, respectively. HMGCS2, 3-hydroxy-3-methylglutaryl-CoA synthase; LCAD, long-chain acyl CoA dehydrogenase; LDH, lactate dehydrogenase; PDH, pyruvate dehydrogenase; VLCAD, very long-chain acyl-CoA dehydrogenase.

In addition to ketone bodies, lactate is another alternative source of energy used by the brain under certain circumstances. Lactate can be transported from the periphery across the BBB by monocarboxylate transporters 1 (MCT1) using facilitated diffusion, and within the brain astrocytes can also serve a source of lactate for neurons (reviewed in Steinman et al., 2016). Neurons express MCT2, which transports lactate intracellularly where lactate dehydrogenase (LDH) converts it into pyruvate, which is then converted to acetyl-CoA by pyruvate dehydrogenase (PDH; Pellerin et al., 2005). Although a direct link has not yet been established in neural tissues, SIRT3 has been shown to deacetylate both LDH (Cui et al., 2015) and PDH in a number of cancer cells (293T, HCT116, HeLa, T47D, MMT and MCF7; Ozden et al., 2014) and mouse skeletal muscle (Jing et al., 2013). These data therefore provide another potential means through which SIRT3 could facilitate the use of alternative energy sources in the brain during times of need. The visual overview of how SIRT3 regulates energy source production and utilization is illustrated in Figure 2.

## The Potential Role of SIRT3 in Neurodegenerative Diseases

Although neurodegenerative diseases manifest with different clinical outcomes, their pathophysiology often converges at the level of mitochondrial dysfunction (Martin, 2012; Morris and Berk, 2015). While there is considerable debate of whether mitochondrial dysfunction is causal to, or consequential of, the progression of these CNS there is general agreement that improving mitochondrial homeostasis and functional capacity could improve outcomes for many degenerative conditions. Thus, elucidating the role of SIRT3 in the progression of these conditions, or for its potential to be targeted to alter disease course, are emerging areas of interest (see Supplementary Table S1).

### SIRT3 in Alzheimer’s Disease

Growing evidence has demonstrated mitochondrial dysfunction is a critical factor contributing to the initiation and progression of AD (Grimm et al., 2016), and recent data suggest SIRT3 may play a role in this process. Expression analysis has revealed changes in *SIRT3* expression levels in post-mortem samples from AD patients and in experimental mouse models. For example, Weir et al. (2012) reported increased SIRT3 mRNA levels in human post-mortem midfrontal and temporal neocortical tissues from AD patients (Weir et al., 2012), while in contrast, Yin et al. (2018) reported decreased SIRT3 mRNA and protein levels in human AD post-mortem cortical regions (Supplementary Table S1). The reason for the discrepancy in SIRT3 expression outcome is unclear, but the results do suggest an AD-related SIRT3 response does occur during the course of the disease.

In mouse models, SIRT3 expression responses have also been identified, but like in AD patients somewhat different outcomes have been observed in different models. *Sirt3* mRNA levels were initially elevated in hippocampal tissues of early symptomatic 6 month-old AD PDAPP mice (which overexpress a human amyloid beta (Aβ) precursor protein (APP) carrying the V717F mutation), but this increase in SIRT3 expression returns to normal levels by 26 months of age (Weir et al., 2012). Both SIRT3 mRNA and protein levels were significantly diminished in APP/PS1 double transgenic mice at 12 months of age (Yang et al., 2015), and decreased SIRT3 expression was similarly found in PSEN1/APP/TAUP301L mice at 24-months of age (Han et al., 2014). The initial up-regulation of SIRT3 may represent an endogenous neuroprotective response early in disease pathogenesis that is then lost as the disease further progresses. Furthermore, over-expression of SIRT3 prevented Aβ-42 induced tau accumulation in cultured cortical neurons derived from mice expressing a human tau transgene, while knockdown of SIRT3 in the same cultured neurons caused an elevation of tau accumulation (Yin et al., 2018). This observation is consistent with that of another recent study which showed treating hamster ovary PS70 cells expressing mutant APP with the SIRT3 activator Honokiol attenuated ROS levels, increased mitochondrial membrane potential, elevated the levels of p-CREB and PGC1α and importantly decreased intracellular levels of Aβ (Ramesh et al., 2018). These results collectively suggest SIRT3 activity may attenuate the over-production and accumulation of tau. While encouraging, further studies will be required to define the contribution and potential translational value of SIRT3 in AD pathogenesis.

### SIRT3 in Parkinson’s Disease

Two agents widely used to induce experimental Parkinsonism disease (PD) in model systems are 1-methyl-4-phenyl-1,2,3,6-tetrahydropyridine (MPTP) and rotenone; both of which are believed to cause PD-like degeneration through their induction of oxidative stress levels and impairment of mitochondrial function. In cultured SHSY-5Y cells, Cui et al. (2017) found that SIRT3 over-expression prevented the ATP loss induced by MPP^+^ (a metabolite of MPTP) treatment, and SIRT3 over-expression also diminished apoptosis, α-synuclein accumulation, and cell death following rotenone treatment of the same cells (Zhang et al., 2016). Consistently, SHSY-5Y cells lacking SIRT3 showed an increase in apoptosis, α-synuclein accumulation, and cell death following the same rotenone treatment (Zhang et al., 2016). In line with the *in vitro* studies, SIRT3-KO mice showed exacerbated degeneration of dopaminergic neurons compared to wild-type mice following MPTP treatment (Liu et al., 2015), and viral-mediated over-expression of SIRT3 protected dopaminergic neurons from α-synuclein-induced degeneration (Gleave et al., 2017). Shi et al. (2017) also observed an increase in oxidative stress and degeneration of SNc dopaminergic neurons from SIRT3-KO mice, which correlated with increased lysine-68 acetylation of MnSOD (MnSODK68). Importantly, SNc dopaminergic neuron degeneration was prevented by either reintroduction of functional SIRT3, or by introduction of a constitutively active deacetylation-mimetic form of MnSODK68 (Shi et al., 2017). Collectively, these studies suggest that the endogenous functions of SIRT3 may indeed play a protective role in PD pathogenesis, and suggest SIRT3 may represent a therapeutic target for PD translational development, particularly since hyperacetylation of MnSODK68 has been observed in post-mortem midbrain tissues from PD patients (Shi et al., 2017).

### SIRT3 in Amyotrophic Lateral Sclerosis

Although there have been few investigations of SIRT3 to date in ALS models, the available data suggest the actions of SIRT3 could influence ALS pathogenesis. As discussed above, (Song et al., 2013) showed the mitochondrial fragmentation and activation of apoptotic cascades in cultured motor neurons derived from SOD1^G93A^ mutant mice could be prevented by the ectopic over-expression of SIRT3. Similarly, Harlan et al. (2016) found that SIRT3 over-expression in SOD1^G93A^ mutant mouse astrocytes rescued the degeneration of co-cultured SOD1^G93A^ mutant mouse motor neurons. Further studies are clearly required, but these results suggest that enhancing SIRT3 activity may provide a protective role in at least for some forms of ALS.

### SIRT3 in Huntington’s Disease

Although the mechanism in neuronal degeneration is not fully understood in Huntington’s disease (HD), mitochondrial involvement has been speculated (Chen, 2011). To date, only one study has examined SIRT3 in the context of HD progression (Fu et al., 2012). Fu et al. (2012) showed that SIRT3 protein levels and deacetylase activity are diminished in a human striatal precursor cell line expressing mutant Htt, and the protective effects observed in these cells following treatment with the stilbenic compound *ns*-(−)-ε-Viniferin (viniferin) were lost when endogenous SIRT3 was knocked down. Although further studies are required, this initial study also suggest SIRT3 up-regulation or enhancement of enzymatic activity could slow the progression of HD.

### The Role of SIRT3 Can Be Cytoprotective or Cytodamaging in Models of Stroke

To date, several studies have examined the role of SIRT3 in different *in vitro* and *in vivo* models of stroke. Kim et al. (2011) reported that the over-expression of SIRT3 in cortical neurons protected against NMDA-excitotoxicity; Dai et al. (2017) demonstrated lentiviral over-expression of SIRT3 protected cultured cortical neurons from an oxygen/glucose deprivation (OGD) challenge; Shulyakova et al. (2014) showed SIRT3 over-expression protected neuronally differentiated PC12 cells from OGD and from apoptotic degeneration induced by trophic withdrawal; Magnifico et al. (2013) found that transient over-expression of full-length mouse SIRT3 protected cultured mouse cerebellar granule neurons from axonal degeneration induced by KCl depletion; and Yang et al. (2017) found that administration of the SIRT3 activator adjudin attenuated glial scar formation and improved functional recovery in mice following transient middle cerebral artery occlusion (MCAO), but that these effects were lost in SIRT3-KO mice. Collectively, these studies support the hypothesis that enhancing SIRT3 prevalence and/or activity can provide protection against a spectrum of insult-related challenges.

However, not all experimental data indicate that the over-expression or increased activation of SIRT3 is beneficial. In fact, quite contrasting outcomes have been observed in some experimental systems. For example, cerebellar granule neurons overexpressing SIRT3 displayed enhanced neuronal death in response to low potassium ion treatment (Pfister et al., 2008), and SIRT3-KO mice displayed smaller brain infarct volumes than wild type mice following reversible MCAO (Novgorodov et al., 2016), and Verma et al. (2018) also recently reported ischemia/reperfusion damage to be reduced in SIRT3-KO mice. In the Novgorodov et al., 2016 study, Sirt3 ablation was found to lessen the activation of ceramide synthases 1, 2 and 6 following reperfusion, which was hypothesized to help preserve the overall function of the challenged mitochondria by decreasing the generation and accumulation of toxic mitochondrial ceramides (reviewed in Novgorodov and Gudz, 2011). The Verma et al. (2018) study employed a similar model, but speculated the observed protection may relate to a compensatory up-regulation of the SIRT1 sirtuin in the brains of SIRT3 KO mice. These results do not necessarily contradict the anti-oxidant or anti-apoptotic capacity of SIRT3, as the severity or type of stroke challenge could differentially activate ROS-dependent or ceramide-dependent cell death cascades (Novgorodov and Gudz, 2011; Mencarelli and Martinez-Martinez, 2013), but they do highlight the possibility that SIRT3 may differentially regulate stroke-related outcomes in tissue- or context-dependent manners (Dittenhafer-Reed et al., 2015).

## Regulation of SIRT3 Expression Levels and Function

Converging data now suggest that augmenting SIRT3 activity may be beneficial in progressive neurodegenerative diseases such as AD and PD, and that modulating SIRT3 activity may also provide protection in different types of stroke depending on context. This section will review current knowledge of endogenous and pharmacological regulators of SIRT3 expression and catalytic activity.

### The Role of Diet and Exercise in Regulating SIRT3 Expression Levels

While the molecular mechanisms remain to be fully elucidated, both diet and exercise have been shown to influence SIRT3 expression levels. Mice maintained on caloric restriction (CR) diets show increased SIRT3 mRNA and protein levels in a host of tissues, which include brown adipose tissue (Shi et al., 2005), skeletal muscle (Palacios et al., 2009; Tauriainen et al., 2011), liver (Tauriainen et al., 2011), adipose tissue (Tauriainen et al., 2011) and brain (Amigo et al., 2017). However, these expression outcomes depend on the degree of CR, as studies also found 48 h of complete fasting decreased *SIRT3* mRNA in human muscle tissue (Edgett et al., 2016). Exercise was also found to increase SIRT3 expression in both rat and mouse striated muscle and brain (Ferrer et al., 2009; Palacios et al., 2009; Hokari et al., 2010; Brandauer et al., 2015; Cheng et al., 2016). Some data have implicated the SIRT3 up-regulation stemming from CR and exercise to arise at least in in part through the AMP-activated protein kinase (AMP-kinase) pathway (Brandauer et al., 2015), as following nutrient restriction and exercise there is an increase in AMP-kinase activity due to elevated AMP/ATP ratios (Richter and Ruderman, 2009; Cantó and Auwerx, 2011). While AMP-kinase has several targets depending on context and cell type, one intriguing target of AMP-kinase is the transcriptional regulator CREB, which is known to activate PGC-1α expression under several conditions (Thomson et al., 2008; Palacios et al., 2009). There may also be convergence on CREB as a regulating factor for SIRT3 expression, as Cheng et al. (2016) found that increases in SIRT3 expression in mouse brain following exercise were abolished by treatment with the NMDA receptor antagonist MK801. These results thereby implicate glutamatergic systems, which are well established to influence activity-dependent CREB signaling (Schurov et al., 1999; Lonze and Ginty, 2002) in exercise-induced SIRT3 expression regulation. This would also be consistent with the elevation of SIRT3 protein levels that were observed in cultured cortical mouse neurons following acute treatment with NMDA *in vitro* (Kim et al., 2011). These collective results therefore suggest both activity-dependent and AMP-kinase-related mechanism could contribute to exercise-related SIRT3 up-regulation in brain, and this up-regulation of SIRT3 underlie some of the general benefits associated with exercise (Radak et al., 2016).

### Regulation of SIRT3 Deacetylase Activity

Accumulating evidence indicates SIRT3 enzymatic activity fluctuates in accordance with the metabolic state of the cell (Shi et al., 2005; Ferrer et al., 2009; Palacios et al., 2009; Hokari et al., 2010; Li et al., 2011; Tauriainen et al., 2011; Dai et al., 2014; Brandauer et al., 2015; Cheng et al., 2016; Amigo et al., 2017). Because SIRT3 uses NAD^+^ as a cofactor for deacetylation reactions, the ratio of NAD^+^/NADH within the mitochondria can influence its overall deacetylase activity. NAD^+^/NADH ratios can be dynamic within cells, particularly in metabolically demanding cells such as neurons (Cantó et al., 2015). As the deacetylation reaction catalyzed by SIRT3 results in the conversion of NAD^+^ into nicotinamide and acetyl-ADP-ribose, high SIRT3 activity could potentially deplete NAD^+^ within mitochondrial microdomains, and thereby limit its own catalytic activity by removing its required co-factor. Moreover, nicotinamide itself is an endogenous inhibitor of the SIRT3 deacetylation reaction (Guan et al., 2014), which suggests the generation of nicotinamide by SIRT3 could feed-back to attenuate its own activity. Finally, it is also intriguing that NAD^+^/NADH ratios tend to increase during metabolic states associated with CR and exercise (Hipkiss, 2008)—which are recognized for their neuroprotective and/or lifespan extension potential (reviewed in Deslandes, 2013; Michan, 2014). In this regard, CR diets increase SIRT3 deacetylase activity in a number of tissues, which include brain (Someya et al., 2010; Amigo et al., 2017), liver (Someya et al., 2010) and inner ear (Someya et al., 2010) and many of the ascribed benefits of both CR and exercise are lost when SIRT3 activity is abrogated (Palacios et al., 2009; Someya et al., 2010; Cheng et al., 2016). These results therefore provide evidence for an intricate link between CR, exercise, increased NAD^+^/NADH ratios, SIRT3 enzymatic activity, and cytoprotective effects associated with exercise and/or CR.

### Genetic Regulation of SIRT3 Expression Levels

Although still a work in progress, several factors that influence the gene and protein expression levels of SIRT3 are beginning to be delineated in specific model systems. At the genomic level, PGC-1α appears to be a key positive regulator of SIRT3 expression (Kong et al., 2010). As such, conditions within cells that induce PGC-1α activity may also induce *SIRT3* gene expression. This may not be surprising, as PGC-1α is well known to activate the expression of a host of mitochondrial and anti-oxidative defense factors (Onyango et al., 2010). However, the mechanisms through which PGC-1α induces SIRT3 expression are complex and may display cell-type specificity. For example, PGC-1α does not appear to bind to the *SIRT3* promoter region directly; rather it induces the expression of other genes whose products partner with PGC-1α and bind to a region of the SIRT3 promoter as heterodimers. Two such target genes activated by PGC-1α are estrogen-related receptor α (ERRα; Kong et al., 2010), and nuclear respiratory factor 2 (NRF2; Mootha et al., 2004). Both ERRα and NRF2 bind to distinct sites located proximal to the *SIRT3* transcriptional start site, and are suggested to be co-dependent on PGC-1α for this binding activity (Kong et al., 2010; Satterstrom et al., 2016). In addition to regulating SIRT3 expression, ERRα and NRF2 also partner with PGC-1α to up-regulate the expression of other nuclear genes encoding mitochondrial factors (Mootha et al., 2004; Eichner and Giguère, 2011). Intriguingly, the activities of at least some of the proteins derived from these genes are also regulated by SIRT3 deacetylase activity once they reach the mitochondria.

### Therapeutic Approaches to Regulate SIRT3 Expression Levels and Function

The activity of SIRT3 can be upregulated by a number of pharmacological (or chemical) compounds. The first substance suggested to increase the activity of specific sirtuin family members was resveratrol (3,4′,5-trihydroxystilbene; RSV; Howitz et al., 2003), a polyphenol found in red wine speculated to increase anti-oxidative defense when consumed in moderate levels (Sun et al., 2010; Singh et al., 2013). Although RSV has been reported to left-shift the Michaelis constant of SIRT1 for substrate (Howitz et al., 2003), its actions on SIRT3 remain more controversial. RSV treatment has been reported to elevate SIRT3 levels in mouse cardiac fibroblasts (Chen et al., 2015) and increase SIRT3 enzymatic activity in HepG2 cells (Desquiret-Dumas et al., 2013). However, RSV treatment was also reported to down-regulate *sirt3* mRNA levels in zebrafish liver (Schirmer et al., 2012), and inhibits human SIRT3 (Gertz et al., 2012). Other RSV-like compounds which are touted to be more stable and potent than resveratrol, such as piceatannol and 4’-bromo-resveratrol (5-(2-(4-hydroxyphenyl)vinyl)-1,3-benzenediol; Gertz et al., 2012; Nguyen G. T. et al., 2013) have inhibitory effects on SIRT3 activity. As discussed above, it is important to note that the activity of SIRT3 is dependent upon NAD^+^ levels, and there is evidence that NAD^+^ levels can be indirectly affected following RSV treatment. For example, Desquiret-Dumas et al. (2013) showed that RSV directly enhances complex I activity, and thereby increases NAD^+^/NADH ratios. The positive regulation of SIRT3 through this resveratrol-NAD^+^ axis is in line with observations that supplementation with the NAD^+^ precursor nicotinamide riboside similarly enhance SIRT3 activity in mammalian cells and mouse tissues (Brown et al., 2014). Thus, it is possible some of the effects associated with resveratrol may stem from indirect effects on NAD^+^ levels.

Other molecules that are structurally distinct from RSV have also been demonstrated to regulate SIRT3 enzymatic activity. Aside from the global sirtuin inhibitor nicotamide (Bitterman et al., 2002), which as discussed above inhibits SIRT3 by competing with NAD^+^ at the SIRT3 cofactor binding site (Guan et al., 2014), some examples of SIRT3 inhibitory modulators are SRT1720 (Jin et al., 2009), 5-amino-2-phenyl-benzoxazole (Salo et al., 2013), LC-0296 (Alhazzazi et al., 2016) and SDX-437 (Patel et al., 2015). Of these, SDX-437 and LC-0296 showed the highest apparent selectivity for SIRT3 over SIRT1 (Patel et al., 2015; Alhazzazi et al., 2016). At present, though, these compounds remain largely untested for their ability to modulate SIRT3 *in vivo*, and specifically within the CNS. With respect to SIRT3 activators, several have been reported, such as oroxylin A (OA; Zhao et al., 2015), viniferin (Fu et al., 2012), HKL [2-(4-hydroxy-3-prop-2-enyl-phenyl)-4-prop-2-enyl-phenol] (Pillai et al., 2015), nitrite (Lai et al., 2016), adjudin (Yang et al., 2017), 7-hydroxy-3-(4′-methoxyphenyl) coumarin (C12; Lu et al., 2017), although similar to what has been observed for inhibitors, the specificity for SIRT3 in *vivo* in the brain remains untested. This is an emerging area, however, with new molecules and analogs being developed rapidly.

## Conclusions and Summary

Taken together, current research sheds light on the widespread role that SIRT3 activity may play in regulating normal brain function, and how its activities could influence the pathogenic course of stroke and different neurodegenerative diseases. Mitochondrial function is cornerstone to maintaining the homeostatic balance of cells, and particularly those with high energetic demands such as neurons.

SIRT3 has now been shown to post-translationally deacetylate and regulate the activity of a number of mitochondrial proteins whose functions are salient for oxidative phosphorylation and for maintaining anti-oxidative defense, and recent work shows SIRT3 is prominently expressed in central nervous system tissue. Given this, it is perhaps surprising that SIRT3 KO mice display only modest physiological or behavioral alterations under baseline conditions (Lombard et al., 2007; Liu et al., 2015). Whether or not exacerbations in behavioral impairments will be more evident under stressful conditions or as a consequence of aging remains largely to be determined, but the targets of SIRT3 that have been identified makes this a plausible possibility. While, SIRT3 deficiency does cause a hyperacetylation of the mitochondrial proteome (Lombard et al., 2007; Cheng et al., 2016), the consequences of SIRT3 over-expression remain less clear, as examinations of the global acetylation state of mitochondria remains to be assessed. Therefore, it is unclear whether the consequences observed in SIRT3 over-expression models stem from global mitochondrial proteome hypoacetylation, or from the regulation of specific SIRT3 targets. There justifiably remains much interest in this specific mitochondrial sirtuin, and while much information has been gleaned, many important questions—particularly within the context of the nervous system—remain to be answered.

## Author Contributions

ES-D conducted literature review, compiled data and wrote the manuscript. RS assembled Tables and proofed the manuscript. JE oversaw project, edited and compiled the final manuscript.

## Conflict of Interest Statement

The authors declare that the research was conducted in the absence of any commercial or financial relationships that could be construed as a potential conflict of interest. The reviewer LET declared a past co-authorship with one of the authors JE to the handling Editor.
