# Supplementary material for: The Role of SIRT3 in the Brain Under Physiological and Pathological Conditions
Source: Front Cell Neurosci. 2018 Jul 25;12:196. doi: 10.3389/fncel.2018.00196 (PMC6068278; doi:10.3389/fncel.2018.00196)
Supplement: Supplementary file 1 [file Table_1.docx]

Supplementary Material

Examining the Influence of SIRT3 in Normal Brain Function and its Potential Role in Neuropathological Conditions

Elena Sidorova-Darmos, Rosa Sommer, James H. Eubanks^*^

*** Correspondence:** Dr. James Eubanks jeubanks@uhnres.utoronto.ca

Supplementary Table 1: SIRT3 and Neurodegenerative Diseases

| **Disease Model** | **Experimental Model** | **Results** | **Reference** |
| --- | --- | --- | --- |
| Alzheimer’s Disease (AD**)** | Midfrontal and Temporal neocortical tissues of human AD patients  AD PDAPP mice overexpressing human APP V717F mutation | Elevated *SIRT3* mRNA levels in AD human tissues  Elevated *Sirt3* mRNA levels early stages (6 month-old mice)  Wild-type levels of *Sirt3* mRNA in late stages (26 month-old mice) | Weir et al., 2012 |
|  | APP/PS1 double transgenic mouse model | Decreased *Sirt3* mRNA and SIRT3 protein levels (12 months) | Yang et al., 2015 |
|  | PSEN1/APP/ TAUP301L mouse model | Decreased *Sirt3* mRNA and SIRT3 protein levels (24 months) | Han et al., 2014 |
|  | Hamster ovary PS70 cells expressing mutant APP  Treated with Honokiol (SIRT3 activator) | Attenuated ROS levels  Increased mitochondrial membrane potential  Elevated p-AMPK, p-CREB, PGC1α protein levels  Decreased total intracellular amyloid beta (Aβ) protein levels | Ramesh et al., 2018 |
|  | Middle temporal gyrus, superior frontal gyrus, primary visual cortex and entorhinal cortex of human AD patients  Primary cortical neurons from  transgenic mice that carry human tau protein (hTau) | Decreased *SIRT3* mRNA and SIRT3 protein levels levels in AD human tissues  SIRT3 knockdown increased total tau expression in hTau mouse cortical neurons  SIRT3 overexpression reduced total tau levels in in hTau mouse cortical neurons | Yin et al., 2018 |
|  | SHSY-5Y cells overexpressing hSIRT3  (MPP+ and rotenone models of PD) | Rescued ATP loss  Diminished cell apoptosis, α-synuclein accumulation, and cell death | Cui et al., 2017  Zhang et al., 2016 |
| Parkinson’s Disease (PD) | SIRT3-KO mice  (MPTP model of PD) | Exacerbated degeneration of dopaminergic neurons degeneration | Liu et al., 2015 |
|  | Viral-mediated hSIRT3 overexpression | Protective against α-Syn-induced dopaminergic neuron degeneration | Gleave et al., 2017 |
|  | SNc dopaminergic neurons from SIRT3-KO mice | SIRT3 overexpression prevented increase in oxidative stress, degeneration and lysine-68 acetylation of MnSOD observed in SIRT3-KO mice | Shi et al., 2017 |
| Amyotrophic Lateral Sclerosis  (ALS) | Cultured motor neurons derived from SOD1^G93A^ mutant mice overexpressing hSIRT3 | Prevents apoptotic cascades and mitochondrial fragmentation | Song et al., 2013 |
|  | SOD1^G93A^ mutant mouse astrocytes over-expressing mSIRT3 co-cultured with motor neurons | Rescued degeneration of SOD1^G93A^ mutant mouse motor neurons | Harlan et al., 2016 |
| Huntington’s Disease (HD) | Human striatal precursor cell line expressing mutant Htt with SIRT3 knockdown | Loss of protective effects of viniferin in absence of SIRT3 | Fu et al., 2012 |
|  | Mouse cortical neurons overexpressing mSIRT3 | Protective against NMDA-induced excitotoxicity | Kim et al., 2011 |
|  | Rat cortical neurons overexpressing SIRT3 | Protected from oxygen / glucose deprivation (OGD) challenge | Dai et al., 2017 |
|  | Neuronally differentiated PC12 cells over-expressing hSIRT3 | Protected from oxygen / glucose deprivation (OGD) challenge  Protected from apoptotic degeneration induced by trophic withdrawal | Shulyakova et al., 2015 |
|  | Cultured mouse cerebellar granule neurons transiently overexpressing mSIRT3 | Protected from axonal degeneration induced by KCl depletion | Magnifico et al., 2013 |
| Stroke | Transient MCAO in SIRT3-KO mice | SIRT3 activator adjudin attenuated glial scar formation and improved functional recovery in WT mice  Protective effects of adjudin lost in SIRT3-KO mice | Yang et al., 2017 |
|  | Rat cerebellar granule neurons overexpressing SIRT3 | Enhanced neuronal death in response to low potassium ion treatment | Pfister et al., 2008 |
|  | Transient MCAO in SIRT3-KO mice | Smaller brain infarct volumes in SIRT3-KO mice  Decreased mitochondrial ceramides following stroke challenge | Novgorodov et al., 2016 |
|  | Transient MCAO in SIRT3-KO mice | Neuroprotection 3 days post-ischemia/perfusion in SIRT3-KO mice | Verma et al., (2017) |
